# The Impact of Preconception TSH on the Reproductive Outcomes of Infertile Women Undergoing the First Fresh D3 Embryo Transfer Cycle

**DOI:** 10.1155/2020/8829138

**Published:** 2020-11-12

**Authors:** Yuchao Zhang, Wenbin Wu, Yanli Liu, Yichun Guan, Xingling Wang, Liting Jia

**Affiliations:** ^1^Department of Reproductive Medicine, The Third Affiliated Hospital of Zhengzhou University, Zhengzhou, Henan, China; ^2^Neonatal Screening Center, The Third Affiliated Hospital of Zhengzhou University, Zhengzhou, Henan, China

## Abstract

**Purpose:**

To investigate the association between high-normal preconception TSH levels and reproductive outcomes in infertile women undergoing the first fresh D3 embryo transfer.

**Methods:**

This was a retrospective study. Euthyroid patients undergoing the first fresh D3 embryo transfer from January 2018 to May 2019 were initially included. The patients were divided into a low-TSH (0.27–2.5 mIU/L) group and a high-normal TSH (2.5–4.2 Miu) group. The reproductive outcomes were compared between the groups.

**Results:**

A total of 1786 women were ultimately included, in which 1008 of whom had serum TSH levels between 0.27 and 2.5 mIU/L and 778 of whom had serum TSH levels between 2.5 and 4.2 mIU/L. The patients were highly homogeneous in terms of general characteristics. High-normal TSH levels had no adverse impact on the clinical pregnancy rate, miscarriage rate, or live birth rate (respectively, aOR = 0.92, 1.30, and 0.88 and *P* = 0.416, 0.163, and 0.219). No significant differences were observed in terms of gestational age, single live birth rates, and birth weight, or birth length.

**Conclusion:**

High-normal TSH levels did not significantly influence reproductive outcomes in infertile women undergoing the first fresh D3 embryo transfer. Further studies are needed to test whether the results might be applicable to a wider population.

## 1. Introduction

Subclinical hypothyroidism (SCH), which was common in clinical practise, was a subtle deficiency in thyroid function regardless of the elevated thyrotropin (TSH) level and normal free thyroxine (FT4) level [1]. There is still debate regarding the definition of SCH (the threshold value might range from 2.5 to 5.5 mIU/L according to different studies) and the decision of when to treat, particularly for women attempting pregnancy [2]. When SCH was defined as a TSH level >4.0 mIU/L, there was fair evidence showing that it was associated with miscarriage and adverse obstetric outcomes. Experts suggested that TSH concentrations <2.5 mIU/L should be maintained during the ART procedure in infertile patients. However, there is insufficient evidence indicating an association between high-normal preconception TSH (2.5–4.0 mIU/L) and the reproductive outcomes [3]. We previously investigated the impact of high-normal preconception TSH on the reproductive outcomes of infertile women undergoing their first IUI treatment and observed no association [4]. Similar results were also observed in women undergoing the first single fresh D5 blastocyst transfer. However, different opinions were held by other instigators as various confounders (such as the transfer of cleavage embryo or blastocysts, the number and quality of the transferred embryo, and the age and ethnicity of included women) influenced the reproductive outcomes [5–14]. For that reason, the aim of this study was to further evaluate the impact of preconception TSH levels between 2.5 and 4.2 mIU/L (which was used as the upper limit of the reference range in our laboratory) on reproductive outcomes in women undergoing their first fresh D3 embryo transfer.

### 1.1. Patients

A total of 2542 infertile women receiving fresh D3 embryo transfer in the Department of Reproductive Medicine, the Third Affiliated Hospital of Zhengzhou University, from January 2018 to May 2019, were initially included. The women underwent standard controlled ovarian hyperstimulation (COH) with a downregulation protocol using a GnRH-agonist or an antagonist protocol according to the patients' conditions. For women with poor ovarian response, mild ovarian stimulation was offered. Oocytes were retrieved transvaginally 34–36 h after HCG administration and were inseminated using either the IVF or the ICSI method. Rescued ICSI was performed when necessary to avoid failure of insemination. Variable cleavage embryos were observed and scored accordingly on the third day after insemination. High-quality embryos were preferentially transferred to the uterus under the guidance of ultrasound.

Patients with the following criteria were excluded: previous embryo transfers (*n* = 361); the presence of overt or subclinical thyroid dysfunction (*n* = 366); and lack of essential data (*n* = 29). After exclusions, 1786 women were transferred with fresh D3 embryos at the first IVF/ICSI cycle and were divided into two groups based on preconception TSH levels (low-TSH group, 0.27–2.5 mIU/L, *n* = 1008; high-normal TSH group, 2.5–4.2 mIU/L, *n* = 778). As euthyroid women with preconception TSH<4.2 mIU/L were included in this study, no further tests or treatments were provided to them after starting the ovarian stimulation. The study has been carried out in accordance with the Code of Ethics of the Declaration of Helsinki.

### 1.2. Laboratory Tests

The measurements of thyroid hormones were performed as previously described [4]. Generally, thyroid function tests were performed no longer than six months before starting the ovarian stimulation protocol. Daily internal quality control and yearly external quality control were carried out by request. The intra-assay coefficients of variation (CV) of serum TSH were 1.3%, and the interassay CV value was 3.5%.

### 1.3. Definition of Clinical Outcomes

Pregnancy was defined as positive serum *β*-hCG (>10 IU/L). Clinical pregnancy was defined as the presence of the gestational sac and fetal heart activity following positive serum *β*-hCG. Early embryo loss was defined as the lack of visible gestational sac or fetal heart activity following positive serum *β*-hCG. Ectopic pregnancy was defined as a pregnancy that did not occur inside the uterine cavity. Miscarriage, which included early abortion, intermediate abortion, and late abortion, was defined as a pregnancy that did not result in delivery. Preterm birth was defined as a gestational age less than 259 days (37 weeks). Live birth was defined as the delivery of live babies. Low live birth weight was defined as the neonatal weight less than 2500 g.

### 1.4. Statistical Analyses

Our primary outcomes were clinical outcomes, such as clinical pregnancy rate, early pregnancy loss rate, miscarriage rate, and live birth rate. The secondary outcomes were obstetric outcomes, such as the single live birth rate, birth weight, birth length, and gestational age. Continuous data are expressed as the mean (SD), and statistical comparisons were performed by either Student's *t* test or the Mann–Whitney *U* test where appropriate. Categorical variables were expressed as numbers or percentages, and the chi-squared test or Fisher's exact test was used for comparisons of categorical variables where appropriate. Binary logistic regression was then used to evaluate the impact of preconception high-normal TSH levels on the clinical and obstetric outcomes. Data were analyzed using SPSS 22.0 statistical software. All *P* values less than 0.05 were considered statistically significant.

## 2. Results

### 2.1. General Characteristics of the Included Women

**Figure 1:**
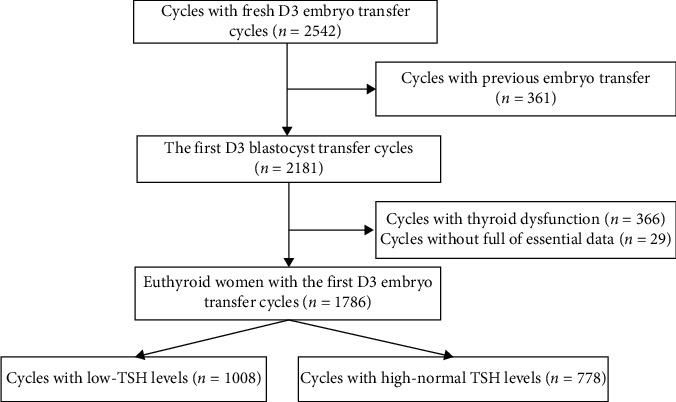
Flowchart of the inclusion of women undergoing the first fresh D3 embryo transfer cycles.

The flowchart of the included women is shown in Figure 1. The results predicted that a cohort of highly homogeneous women was included. Specifically, there were no differences between the TSH groups in terms of age, BMI, AMH, FT4, endometrial thickness, duration of infertility, and basal FSH (Table 1). Furthermore, there were no differences in the frequencies of the causes or type of infertility between the two groups (*P* = 0.107, 0.126). The frequencies of insemination methods and number and high-quality of transferred embryos were similar in both the groups (*P* = 0.172, 0.669, and 0.298, respectively). No difference was noted in terms of the scheme of ovarian stimulation (*P* = 0.355).

### 2.2. Clinical Outcomes of the Women Undergoing the First Fresh D3 Embryo Transfer

As shown in Table 2, comparisons of pregnancy rate, clinical pregnancy rate, live birth rate, and single live birth rate were performed between the low-TSH and high-normal TSH groups. There were no significant differences in the abovementioned reproductive outcomes between the two groups. In addition, there were no significant differences in the early pregnancy loss rate, miscarriage rate, or ectopic pregnancy rate between the two groups. Furthermore, binary logistic regression analysis between the groups indicated the number of high-quality D3 embryos was positively associated with the clinical pregnancy and live birth, and that was negatively associated miscarriage with adjusted ORs of 1.73 (*P* < 0.001), 1.76 (*P* < 0.001), and 0.66 (*P* = 0.002), respectively, while younger age had slightly but significantly positive effects on the clinical pregnancy, live birth, and miscarriage with adjusted ORs of 0.93 (*P* < 0.001), 0.91 (*P* < 0.001) and 1.12 (*P* < 0.001), respectively. Anyhow, high-normal TSH levels had no adverse impact on the abovementioned outcomes (aOR = 0.92, 0.88, and 1.30 and *P* = 0.416, 0.219, and 0.163 for clinical pregnancy rate, live birth rate, and miscarriage rate, respectively) (Table 3).

### 2.3. Obstetric Outcomes of the Women Undergoing the First Fresh D3 Embryo Transfer

We further compared obstetric outcomes between the low-TSH and high-normal TSH groups. Of the 1786 women included, 471 in the low-TSH group had live births, while 385 in the high-normal TSH group had live births (Table 4). No significant difference was observed in terms of the number of live birth (*P* = 0.129). The preterm birth rates were similar between the two groups (singleton, *P* = 0.065; twin, *P* = 1.000). The low-weight birth rates were also similar between the two groups (singleton, *P* = 0.679; twin, *P* = 1.000). In addition, the gestational age and birth length of single live births were similar between the low-TSH and high-normal TSH groups (*P* = 0.621 and 0.943, respectively), as were the gestational age and length of twin live births (*P* = 0.157 and 0.904, respectively).

## 3. Discussion

In this retrospective study, we investigated the association between preconception TSH and the reproductive outcomes of 1786 infertile women undergoing the first fresh D3 embryo transfer at a university-affiliated hospital and found that a high-normal preconception TSH level (between 2.5–4.2 mIU/L) was not associated with lower odds of clinical pregnancy and live birth or higher odds of miscarriage. Neonatal outcomes were also comparable between low-TSH and high-normal TSH groups.

Chen et al. [15] investigated the association of preconception TSH and clinical outcomes in a Chinese woman conceived naturally and clarified that preconceptionally high-TSH levels were associated with a small but significantly increased risk of overall adverse events, even within the normal unpregnant range, and concluded that TSH <2.5 mIU/L was more suitable for the assessment of women planning for a pregnancy. However, the impact of preconception TSH in women undergoing fertility treatment is conflicting.

Our previous results were in agreement with other studies, which demonstrated that mildly elevated preconception TSH levels before treatment had no adverse impact on the clinical outcomes in infertile women undergoing intrauterine insemination (IUI) treatment [4]. In addition, we further included a cohort of highly homogeneous infertile women who were transferred with a single fresh D5 blastocyst and found similar reproductive outcomes between the low-TSH and high-normal TSH groups (data not shown). Further on, another cohort of homogeneous infertile women was included and underwent the first D3 embryo transfer in this study. Again, similar results were observed regardless of different embryo transfer schemes and possible inclusion of infertile populations with different age, ovarian reserve, and ovarian stimulation protocol.

Similarly, Reh et al. used different TSH cutoffs in a large retrospective cohort study which included first-cycle IVF patients, and no differences in the rates of clinical pregnancy, delivery, or miscarriage were observed after adjustments were made for age [16]. Aghahosseini et al. reported a lack of association between TSH levels in the range of 0.5–4.5 mIU/L and clinical pregnancy rate and stated that lowering the upper limit of normal TSH should still be considered a scientific debate [6]. Chai et al. also reported that the clinical pregnancy rate and miscarriage rate were not impaired when the TSH level was high-normal [8]. In agreement with our results, Unuane et al. and So et al. both claimed that cumulative pregnancy rates were similar between women with low-TSH levels and those with high-normal TSH levels [5, 14].

According to the molecular basis of TSH and thyroid hormone action during implantation and early development, thyroid hormone might be involved in endometrium preparation for pregnancy and initial trophoblast development, and thyroid hormones are essential players in the mechanisms regulating implantation and early fetal development [17]. As a matter of fact, FT4 levels between low-TSH and high-normal TSH groups were similar in either our previous studies or the study performed by Unuane et al. [14], which may partially explain the similar results between the two groups.

In contrast, Diana et al. claimed a detrimental effect of high-normal preconception TSH levels on the clinical outcomes of infertile women undergoing their first IVF cycle [10]. Discrepancies might be explained by the differences in the included population and the transferred embryos. Specifically, the proportion of women in the high-normal TSH group was much higher in our study (43.5% vs. 18.3%). Notably, the prevalence of SCH (TSH cutoff: 4.2 mIU/L) in the general population in China was 16.7% [18], which was close to the proportion of the population with high-normal TSH levels in the study by Diana et al. In addition, the women in our study were transferred with fresh D3 embryos, while details about the transferred embryos in the study by Diana et al. were not reported. Valerie et al. further demonstrated that a preconception TSH level >2.5 mIU/L was associated with a lower gestational age at delivery and lower birth weight in women undergoing IVF treatment [19]. It should be noted that the study performed by Valerie et al. included a population with TSH level >4.0 mIU/L, which was currently used as the cutoff to define SCH, while our study only included women with TSH values lower than the upper limit of the reference range. The reproductive outcomes which included the incidence of preterm birth rate and low-weight birth rate were comparable when we controlled for twin live births, which supported our observations found in women undergoing their first IUI treatment.

There were also some limitations in this study. First, this was a retrospective study with data collected from a single center, and selection bias was inevitable. Second, we did not report the status of thyroid antibodies in infertile women, which might have a role in infertility and miscarriage. However, on the one hand, the existing data are controversial regarding whether thyroid antibodies are associated with infertility or adverse reproductive outcomes [7, 8, 12, 14, 20]. On the other hand, according to the two RCTs of high quality, euthyroid women with positive TPO did not benefit from treatment with LT4 [21, 22]. Third, we included a cohort of infertile women with specified conditions, which we believed to be one of the crucial factors explaining the discrepancies in the results. Whether the results might be applied to women with repeated embryo transfer or healthy women remains to be tested. Finally, we did not monitor the TSH level once it was within the normal range before treatment. It was confirmed in other studies that high-normal TSH was more prone to increase to a level higher than the upper limit of normal range during ovarian stimulation and decrease within the normal range after embryo transfer [23–27]. Whether temporarily increased TSH levels might affect clinical outcomes remained unknown. The strength of our study was the highly homogeneous population-based cohort of euthyroid women, as the number, stage, or quality of transferred embryos, as well as the age and ovarian reserve, might potentially affect the results of embryo transfer. We also compared the differences in obstetric outcomes, such as gestational age, birth weight, and birth length, between the low-TSH and high-normal TSH groups and found that, regardless of TSH levels within the normal range, age and number of high-quality D3 embryos were independently associated with reproductive outcomes such as clinical pregnancy rate, live birth rate, and miscarriage rate.

## 4. Conclusion

We included infertile women as homogeneous as possible to minimize the bias that may lower the power of the impact of TSH on reproductive outcomes. We found that a high-normal TSH level did not significantly influence the reproductive outcomes of infertile women undergoing the first fresh D3 embryo transfer. Further studies are needed to test whether the results might be applicable to a wider population.

## Figures and Tables

**Table 1:** The general characteristics of the included women based on TSH levels.

	Low-TSH (0.27–2.5mIU/L)	High-normal TSH (2.5–4.2mIU/L)	*P*
*N*	1008	778	
Age (years)	31.82 (4.75)	31.62 (4.59)	0.371
BMI (kg/m^2^)	23.71 (3.27)	23.98 (3.38)	0.088
AMH (pmol/L)	25.62 (21.03)	27.17 (23.65)	0.143
FT4 (pmol/L)	16.77 (6.81)	16.51 (5.56)	0.385
FT3 (pmol/L)	4.78 (0.72)	4.83 (0.73)	0.156
FSH (IU/L)	7.52 (2.32)	7.67 (2.3)	0.169
Endometrial thickness (mm)	11.29 (2.31)	11.46 (2.37)	0.138
Duration of infertility (year)	3.52 (2.68)	3.68 (2.78)	0.212

*Insemination methods of transferred embryos*			
ICSI	246 (24.4%)	214 (27.5%)	0.172
IVF	752 (74.6%)	552 (71%)	
IVF/ICSI	10 (1%)	12 (1.5%)	

*Ovarian stimulation protocol*			
GnRH agonist	915 (90.8%)	720 (92.5%)	0.355
GnRH antagonist	88 (8.7%)	56 (7.2%)	
Mild ovarian stimulation^a^	5 (0.5%)	2 (0.3%)	

*Causes of infertility*			
Male factors (azoospermatism)^b^	171 (17%)	159 (20.4%)	0.107
Male factors (others)	56 (5.6%)	46 (5.9%)	
Female mixed factors	38 (3.8%)	42 (5.4%)	
Ovulation failure	70 (6.9%)	53 (6.8%)	
Pelvic and fallopian tube factors	345 (34.2%)	259 (33.3%)	
Factors on both sides	257 (25.5%)	160 (20.6%)	
Unexplained	71 (7%)	59 (7.6%)	

*Type of infertility*			
Primary	545 (54.1%)	392 (50.4%)	0.126
Secondary	463 (45.9%)	386 (69.6%)	

*Number of transferred embryos*			
1	110 (10.9%)	80 (10.3%)	0.669
2	898 (89.1%)	698 (89.7%)	

*Number of high-quality transferred embryos*			
0	150 (14.9%)	137 (17.6%)	0.298
1	258 (25.6%)	193 (24.8%)	
2	600 (59.5%)	448 (57.6%)	

^a^the couples with mild ovarian stimulation were of poor ovarian response. Generally, those couples would have the embryos vitrified for subsequent frozen-thawed transfer cycle; ^b^ the couples with infertile cause of male factor (azoospermatism) were provided with donor sperm for insemination.

**Table 2:** Clinical outcomes after the first fresh D3 embryo transfer.

	Low-TSH (0.27–2.5 mIU/L) (*n* (%))	High-normal TSH (2.5–4.2mIU/L) (*n* (%))	*P*
Pregnancy rate	606 (60.1)	476 (61.2)	0.648
Clinical pregnancy rate	568 (56.3)	452 (58.1)	0.459
Live birth rate	471 (46.7)	385 (49.5)	0.227
Early pregnancy loss rate	38 (6.3)	24 (5)	0.388
Miscarriage rate	87 (15.3)	56 (12.4)	0.181
Still birth rate	2 (0.4)^a^	1 (0.2)	1.000
Ectopic pregnancy rate	10 (1.8)	10 (2.2)	0.605

^a^: two women conceived two babies but gave birth to a singleton.

**Table 3:** The possible factors that impact on clinical outcomes.

	Clinical pregnancy		Live birth		Miscarriage	
	aOR (95%CI)	*P*	aOR (95%CI)	*P*	aOR (95%CI)	*P*
TSH (low VS high-normal)	0.92 (0.76, 1.12)	0.416	0.88 (0.73, 1.08)	0.219	1.30 (0.90, 1.88)	0.163
Age	0.93 (0.91, 0.95)	<0.001	0.91 (0.89, 0.93)	<0.001	1.12 (1.08, 1.17)	<0.001
AMH	1.00 (0.96, 1.01)	0.068	1.00 (0.98, 1.01)	0.269	1.01 (0.98, 1.01)	0.181
Number of high-quality embryos	1.73 (1.51, 1.99)	<0.001	1.76 (1.52, 2.03)	<0.001	0.62 (0.51, 0.86)	0.002
Number of transferred embryos	1.34 (0.96, 1.88)	0.085	1.31 (0.91, 1.87)	0.136	1.03 (0.53, 1.99)	0.924

**Table 4:** The obstetric outcomes of the women undergoing the first fresh D3 embryo transfer.

		Low-TSH group (*n* = 471)	High-normal TSH group (*n* = 385)	*P*
Number of live births	Singleton	312 (62.2%)	274 (71.2%)	0.139
	Twin	159 (33.8%)	111 (28.8%)	

Birth weight (g)	Singleton	3345 (575.7)	3390 (545.2)	0.334
	Twin	2527.9 (479.1)	2566.5 (445.1)	0.342

Birth length (cm)	Singleton	50.2 (2.2)	50.2 (2.3)	0.943
	Twin	48.1 (2.7)	48.1 (2.6)	0.904

Gestational age (weeks)	Singleton	38.9 (1.8)	39 (1.6)	0.621
	Twin	36.2 (2.2)	36.5 (1.6)	0.157

Preterm birth	Singleton	36 (11.5%)	19 (6.9%)	0.065
	Twin	73 (45.9%)	51 (45.9%)	1.000

Low-weight birth	Singleton	14 (4.4%)	10 (3.6%)	0.679
	Twin	51 (32.0%)	35 (31.5%)	1.000

## Data Availability

All data generated or analyzed during this study are included in this published article.
